# Evaluation of the Sealing Ability of Three Obturation Techniques Using a Glucose Leakage Test

**DOI:** 10.1155/2017/2704094

**Published:** 2017-06-19

**Authors:** Katarzyna Olczak, Halina Pawlicka

**Affiliations:** Department of Endodontics, Medical University of Lodz, Pomorska 251, 92-213 Lodz, Poland

## Abstract

The aim of this study was to evaluate the sealing ability of three different canal filling techniques. Sixty-four roots of extracted human maxillary anterior teeth were prepared using ProTaper® rotary instruments. The specimens were then randomly divided into 3 experimental groups (*n* = 16) and 2 control groups (*n* = 8). The root canals were filled using cold lateral compaction (CLC group), continuous wave condensation technique using the Elements Obturation Unit® (EOU group), and ProTaper obturators (PT group). For the negative control group, 8 roots were filled using lateral compaction as in the CLC group, and the teeth were covered twice with a layer of nail varnish (NCG group). Another 8 roots were filled using lateral compaction, but without sealer, and these were used as the positive control (PCG group). A glucose leakage model was used for quantitative evaluation of microleakage for 24 hours and 1, 2, 3, 4, 5, 6, 7, 8, 9, 10, 11, and 12 weeks. No significant difference in the cumulative amount of leakage was found between the three experimental groups at all observation times. The lateral condensation of cold gutta-percha can guarantee a similar seal of canal fillings as can be achieved by using thermal methods, in the round canals.

## 1. Introduction

Adequate obturation of the root canal system constitutes one of the most important stages of endodontic treatment, which significantly affects its final result [1]. Even a correctly selected sequence of irrigants, the use of additional passive ultrasonic activation, and modern techniques of mechanical root canal preparation are not capable of eliminating all oral cavity microorganisms. When the root canal filling is not tightly sealed, tissue fluids or saliva components are excellent mediums for bacteria [2–4]. Nowadays, root canals are mainly filled with gutta-percha combined with a small amount of sealer, using cold or warm gutta-percha methods [1, 5–7]. Two sets of techniques for filling root canals with gutta-percha exist: solid core or “cold gutta-percha” and softened core or “warm gutta-percha”/thermal methods. The “cold gutta-percha” techniques include the single-cone technique and lateral compaction/condensation. The most commonly used warm gutta-percha methods are warm vertical compaction, sometimes performed with various modifications, continuous wave technique, injection-molded gutta-percha, and core carrier (thermoplasticized obturator) technique [1, 8]. The contemporary single-cone obturation technique uses larger master cones (greater taper) that best match the geometry of canals prepared with nickel-titanium rotary instruments. The use of these gutta-percha points does not require any accessory points or lateral condensation. Lateral compaction (condensation) techniques need one master cone and many additional secondary points. After cementing the master cone, special instruments, spreaders, are placed in the canal, and the master cone is laterally compacted against the walls of root canals. A spreader is then removed and the first accessory point inserted into the canal. The procedure is repeated until it is not possible to insert another gutta-percha cone further than 2 mm to 3 mm into the root canal [1, 8]. Cold lateral compaction is regarded as the benchmark against which other obturation techniques are evaluated. Many studies have examined the quality of the fillings obtained by using lateral condensation and the thermal method. In the early 1960s, Dr. Herbert Schilder created the warm gutta-percha vertical condensation technique. The purpose of this method is to obturate the canal with a filling material softened by heat and packed by vertical pressure, from the coronal to the apical part of the root. The master cone is plasticized with a hot instrument of various specialized equipment systems (e.g., System B®). Once the down-pack is complete, reverse waves of condensation are carried out to complete the backpack [8]. About twenty years later, in 1996, Buchanan created the continuous wave of condensation obturation technique (CWT), which was a modification of Schilder's warm vertical condensation [9]. The main difference between these two techniques is that a heat plugger (in the CWT method) is initially placed at the first introduction through the master cone to within 3 to 5 mm of the working length. In the original warm vertical condensation method described by Schilder, the plugger is introduced several times until it reaches 3 to 5 mm from the apex. In continuous wave of condensation method, the middle and coronal thirds of the canal are then “backfilled” using a gutta-percha injection technique. In the injection technique, gutta-percha is thermoplastically molded and ejected from a needle into the canal [9]. In order to facilitate the filling procedure of CWT, manufacturers offer different devices in which the instrument to plasticize gutta-percha in the tooth is combined with a gutta-percha injection syringe (e.g., Elements Obturation Unit, BeeFill 2 in 1®, Calamus®). A slightly different method of filling root canals is the core carrier (thermoplasticized obturator) technique. This obturation technique was designed and presented in 1978 by Johnson. Initially, this system relied on metallic carriers coated by a layer of gutta-percha, intended to be heated over an open flame. Contemporary obturators are made of radiopaque plastic or a cross-linked gutta-percha central carrier surrounded by a layer of gutta-percha. After heating in a special oven, the obturator is placed into the canal. Next, the coronal part of the obturator is removed [10].

The sealing of the fillings has been often discussed in scientific literature. However, it is difficult to indicate the predominance of one technique over the other [11–15]: despite the wealth of research on this subject, no unequivocal consensus has been reached [16–22]. A number of studies report no differences in tightness of fillings after the application of different methods, and little difference is noted between cold and warm gutta-percha techniques [16, 17]. Some studies report that fillings created using thermal methods demonstrate better tightness than those using cold gutta-percha [18, 19], while others suggest the opposite [20, 21].

Hence, no clear consensus exists on the efficacy of root canal filling methods. With this in mind, the present study evaluates the sealing of root canal fillings performed using warm and cold gutta-percha techniques. Various experimental models have been described to determine leakage along root canals filled [17, 19–21]. Although dyes, radioisotopes, or bacteria penetration techniques have been used to evaluate the seal of endodontic materials, the glucose leakage test/glucose leakage model (GLM) has been advocated as more clinically relevant [22]. The Discussion describes the process of checking the seal of the canal filling in greater detail.

Hence, the aim of the present study was to compare the sealing ability of three endodontic filling methods (cold lateral condensation technique, continuous wave condensation technique, and thermoplasticized obturator technique) using the glucose leakage test. The null hypothesis is that no difference exists between the seals of root canals filled with these three endodontic filling techniques.

## 2. Materials and Methods

Preparation of root specimens for laboratory tests: the experiment was approved by the Bioethics Committee of the Medical University of Lodz (number RNN/129/08/KE). Sixty-four roots of extracted human maxillary anterior teeth were prepared using ProTaper rotary instruments. The teeth were randomly assigned to 5 groups: 3 study groups (16 teeth each) and 2 control groups with 8 teeth in each. The roots were selected for the study based on the following criteria:

- No previous root canal treatment
- No visible signs of root damage in the form of caries, resorption, or root fracture
- The presence of only one straight, round root canal
- Fully developed root apices
- The diameter of the physiological foramen not larger than a size 15 K file

The teeth were stored in aqueous solution of 0.2% sodium azide (NaN_3_). Prior to canal instrumentation, the roots were shortened to the same working length of 11.5 mm. The root canals were chemomechanically prepared with engine-driven ProTaper rotary 6 instruments (Dentsply Maillefer®, Ballaigues, Switzerland) and irrigated with 5,25% NaOCl. A F3 finishing file was the last instrument used in the apical region. After preparation, the root canals were flushed with 15% EDTA, 5,25% NaOCl, and 0,9% NaCl and dried with paper points. Next the root canals were filled in the following way.

*Group 1* (cold lateral condensation technique (group CLC)). First, the master cone was adjusted to reach the working length and a slight “tug back” was felt at its withdrawal. Lateral condensation was performed with a #25 nickel-titanium spreader and #20 accessory gutta-percha cones. 

*Group 2* (continuous wave condensation technique using an Elements Obturation Unit (group EOU)). The vertical condensation warm gutta-percha method was performed using the System B tip of the Elements Obturation Unit and a ProTaper gutta-percha cone of F3 size. The heated System B tip was introduced into the canal to a depth 3 mm shorter than the determined working length. The remaining middle and coronal parts of the canal were filled with injected warm gutta-percha delivered from the Elements Obturation Unit Extruder. 

*Group 3* (ProTaper obturators (group PT)). ProTaper obturators #F3 (Dentsply Maillefer, Ballaigues, Switzerland) were used to fill the root canals of the third group. 

*Group 4* (a negative control group (NCG)). The canals were filled with the cold lateral condensation technique in a similar way to group 1. Prior to the commencement of glucose leakage test, the teeth in the negative control group were covered twice with a layer of nail varnish. 

*Group 5* (positive control group (PCG)). Root canals were filled using the cold gutta-percha lateral condensation technique without sealer. After condensation was complete, the gutta-percha cones were severed with a heated excavator, but not additionally condensed with a plugger.

In groups 1, 2, 3, and 4, AH Plus® material (Dentsply Maillefer, Ballaigues, Switzerland) was used as sealer. After canal obturation, all the roots were stored in test tubes wrapped in sterile gauze pads saturated with of 0.1% NaN_3_ aqueous solution. The specimens were kept in an incubator at 37°C and 100% humidity for 3 weeks.

### 2.1. Construction of the Glucose Leakage Test Device

**Figure 1:**
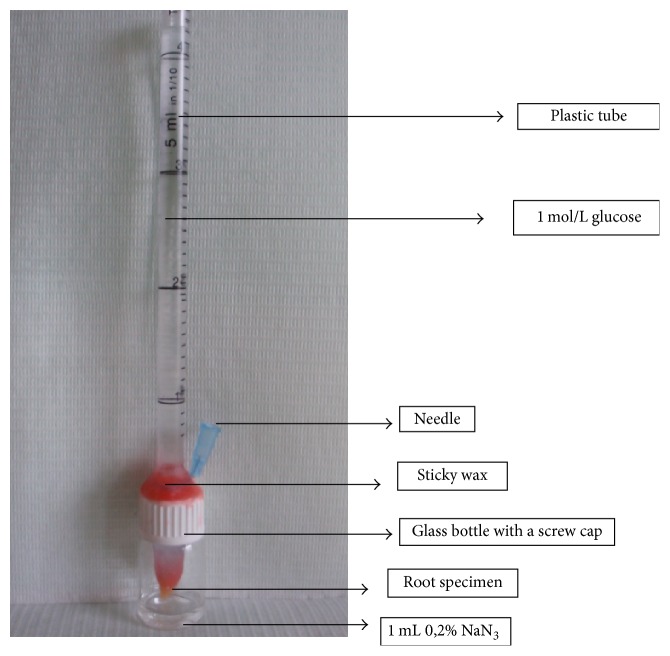
Glucose penetration model.

Each root was mounted in a leakage device, as described by Xu et al. [22]. A separate model made of single elements was constructed for each tooth sample (Figure 1). The external surface of each tooth, apart from the root apex, was coated with wax. Teeth from the negative control group were coated with two layers of nail varnish and then with wax before the experiment.

After tight attachment of all device elements, 1 mol/L glucose solution was added as a tracer containing 0.2% NaN_3_ to inhibit the growth of decomposing glucose bacteria. The mixture was injected into the device through a plastic tube until the top of the solution was 14 cm higher than the coronal orifice of the root, which created a hydrostatic pressure of 1.5 kPa (15 cm of H_2_O). Following this, 5 mL of the glucose solution containing 0.2% NaN_3_ was added to each system. The glucose solution was in the plastic tube and the Eppendorf vial. Using a pipette, 1 mL of 0.2% NaN_3_ was placed into the glass bottle, in which the root apex of the tooth tested was immersed. Glucose, which could pass only through the canal filling, was collected in NaN_3_ in the glass bottle. To determine the loss of sodium azide during the experiment, an additional system without the tooth was prepared, in which the Eppendorf vial bottom was not removed. This system was weighed on high-precision laboratory scales (Analytical Balance, Radwag®). All the systems were stored in an incubator at 37°C and 100% humidity. The specimens were stored according to Shemesh et al. [23] with some modifications. Prior to their placement in an incubator, all the systems were inserted into a hermetically closed container housing cups with distilled water. Thus, the risk of the volume of the glucose and sodium azide solutions being decreased by evaporation was reduced. During the experiment, the system without the tooth was weighed to allow the potential evaporation of the solutions to be accounted for. Furthermore, the level of the glucose solution in plastic tubes was checked.

### 2.2. Glucose Concentration Measurements

In order to determine the glucose concentration, 10 *μ*L of the solution was taken from each glass bottle after 1 day and then at 7-day intervals for the subsequent 12 weeks. When the solution was collected, 10 *μ*L of fresh 0.2% NaN_3_ was added to the bottle to maintain a constant volume of liquid (1 mL). To evaluate the glucose concentration, 1 mL of Glucose-Reagent for determining glucose concentration (BioMaxima®) was added to the vial. Upon addition, the glucose contained in the test tube was oxidized by glucose oxidase to gluconic acid and hydrogen peroxide. In the presence of peroxidase, the hydrogen peroxide reacts with phenol and 4-aminoantipyrine (4-AA) to form a coloured compound, quinoneimine. The intensity of its colour is directly proportional to the glucose concentration. The reaction proceeded according to the scheme:

(1) D-glucose+H2O+O2⟶gluconic  acid+H2O2H2O2+4-AA+phenol⟶quinoneimine+4H2O

After 10 minutes of incubation at 25°C, a Beckmann DU-600® spectrophotometer was used to read the absorbance of the standard specimen and the tested specimens at 490 nm.

### 2.3. Statistical Analysis

The results were analysed to determine whether any statistically significant differences existed between the studied techniques regarding their ability to seal the canal fillings. The Kruskal-Wallis test was used to compare the amount of glucose (concentration) that leaked along the canal filling at different time points. The chi-square test was used to determine and compare the particular techniques of the pulp cavity obturation with regard to the number of specimens in which glucose leakage was observed. Values below *p* = 0.05 were regarded as statistically significant. All calculations were performed using Statistica 10 software.

## 3. Results

**Figure 2:**
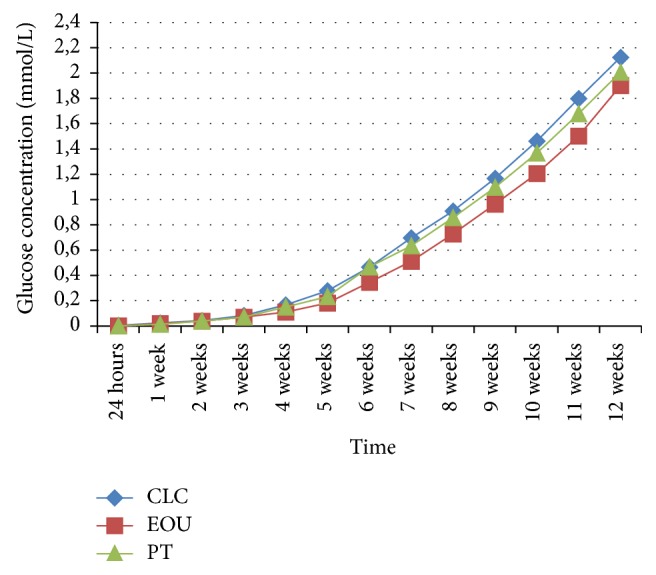
Mean glucose concentrations in mmol/L in CLC, EOU, and PT group throughout the experimental period (CLC, cold lateral condensation technique; EOU, continuous wave of condensation technique using Elements Obturation Unit; PT, ProTaper obturators).

**Figure 3:**
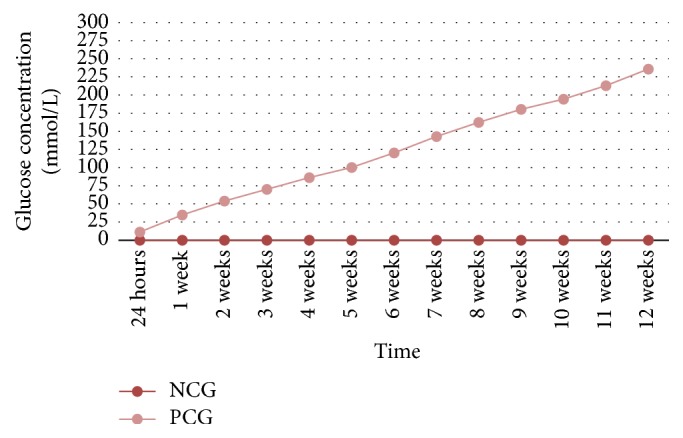
Mean glucose concentrations in mmol/L in control groups throughout the experimental period (NCG, negative control group; PCG, positive control group).

**Figure 4:**
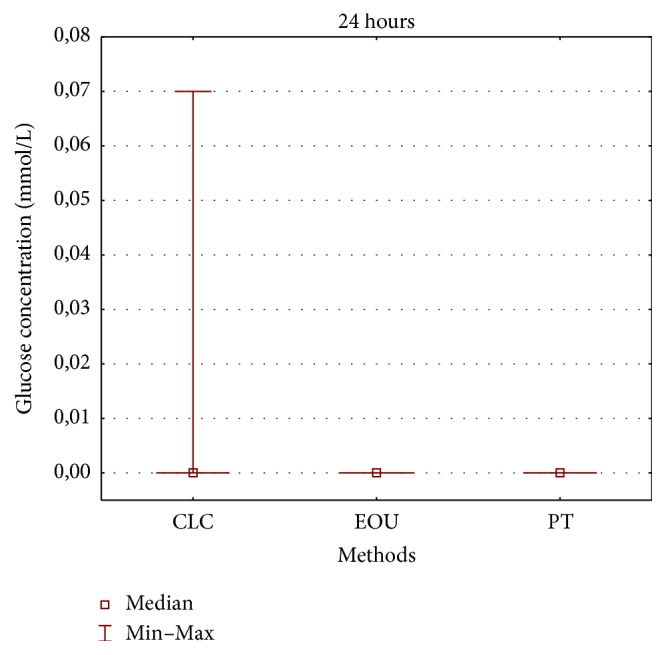
Glucose concentrations in mmol/L in CLC, EOU, and PT group after 24 hours (CLC, cold lateral condensation technique; EOU, continuous wave of condensation technique using Elements Obturation Unit; PT, ProTaper obturators).

**Figure 5:**
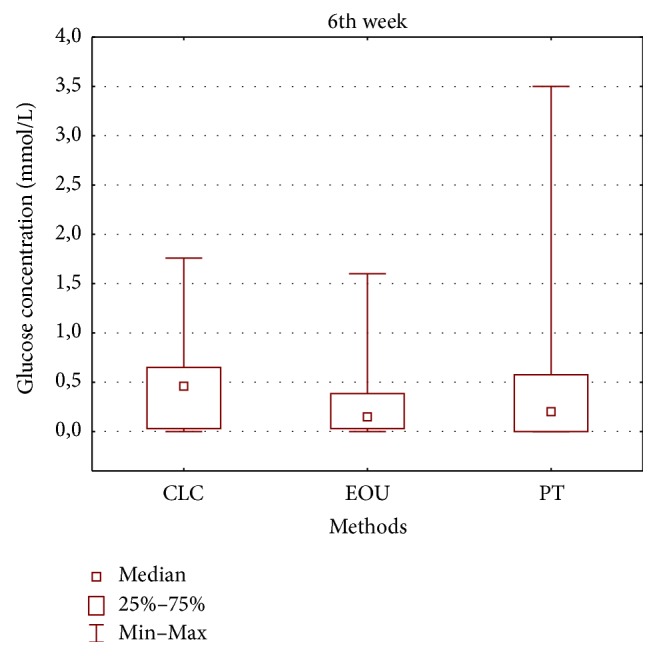
Glucose concentrations in mmol/L in CLC, EOU, and PT group at the 6th week (CLC, cold lateral condensation technique; EOU, continuous wave of condensation technique using Elements Obturation Unit; PT, ProTaper obturators).

**Figure 6:**
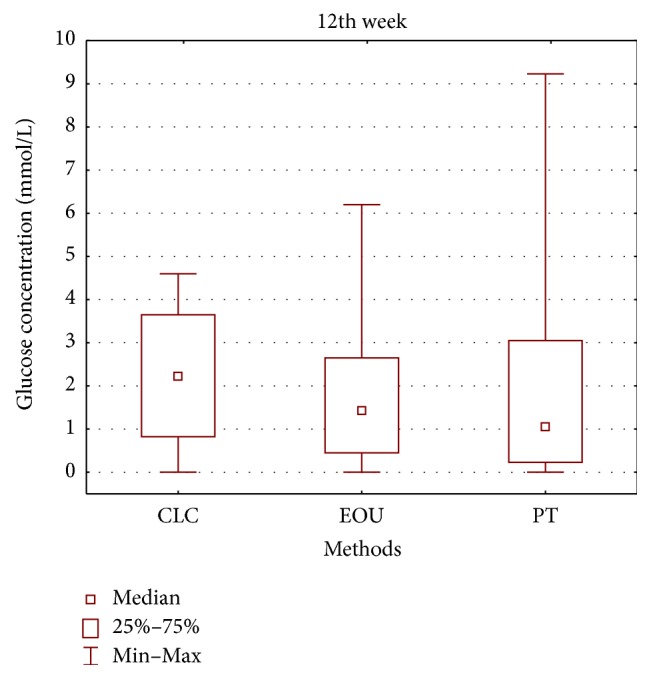
Glucose concentrations in mmol/L in CLC, EOU, and PT group at the 12th week (CLC, cold lateral condensation technique; EOU, continuous wave of condensation technique using Elements Obturation Unit; PT, ProTaper obturators).

**Figure 7:**
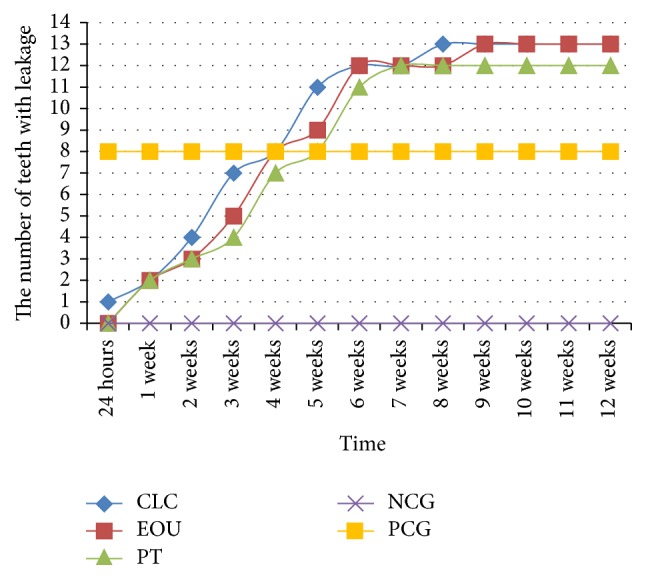
The number of specimens with detectable leakage in each group (CLC, cold lateral condensation technique; EOU, continuous wave of condensation technique using Elements Obturation Unit; PT, ProTaper obturators; NCG, negative control group; PCG, positive control group).

During the experiment, an increase was observed in the glucose leakage in the fillings of the teeth from three study groups and the positive controls (Figures 2 and 3). In the negative control group, no glucose leakage was found in any evaluated root canal filling, while the highest glucose leakage was detected in the positive controls (Figure 3). Among the three obturation techniques evaluated in the study, the highest glucose concentration was noticed in the group of teeth filled with the lateral condensation technique of cold gutta-percha (Figure 2). However, no statistically significant difference was observed between the applied pulp cavity obturation methods with regard to the sealing ability of canal fillings (*p* > 0.05) (Figures 4–6). Therefore, the null hypothesis was accepted. Moreover, in each study group, the number of teeth with glucose leakage also increased with time (Figure 7). However, no statistically significant difference was observed between the pulp cavity obturation techniques with regard to the number of unsound canal fillings (*p* < 0.05).

## 4. Discussion

As one of the priorities of endodontic treatment is to obtain excellent quality root canal fillings, the seal of the fillings has been evaluated by many studies, most of these being performed in vitro. However, as the nature and amount of leakage observed in in vitro penetration models cannot be directly applied to the clinical situation, some researchers question the validity of their use [24, 25]. Nevertheless, these in vitro studies provide valuable information, and when properly selected, prepared, and interpreted, they can contribute to a proper assessment of treatment methods. It is not always possible to evaluate materials under clinical conditions using in vitro approaches, even if for ethical reasons. In addition, before a medical device or instrument is tested in vivo, it must be evaluated in advance under laboratory conditions. To obtain the highest value, in vitro experiments must be properly selected to reproduce the clinical situation as accurately as possible. Dye penetration tests should be avoided, as better and more accurate methods for assessing the seal of fillings are available [22, 24, 25]. Since 2005, the glucose leakage test has been the most frequently applied method of evaluating the quality of filling seals [22]. Its key advantage is that glucose alone is used as the tracer. Glucose is a hydrophilic substance of low molecular weight, which allows it to penetrate the spaces available for toxins and bacterial enzymes. As bacteria feed on glucose, there is a direct relationship between the amount of available sugar, the number of harmful microorganisms, and the effect of endodontic treatment [22]. Another advantage of the glucose test is the fact that it allows the seal of root canal fillings to be traced for a long period of time without the need to destroy the specimen, as it happens in the dye penetration test [22]. Furthermore, as the glucose solution cannot penetrate through the root dentine, test results cannot be false positives [23]. In addition, no chemical reaction is induced between the solutions used in the glucose leakage test and the pulp cavity obturation materials applied in the present study [26]; this is an important point because the glucose test cannot be used to assess the seal of all fillings: for example, it is not advisable to use GLM to evaluate materials with Ca(OH)_2_ because Ca(OH)_2_-containing products react directly with glucose [26]. Furthermore, the GLM is quite a complicated test that requires extraordinary discipline and is time-consuming: each tooth must be prepared with a separate arrangement of individual elements which must be very carefully and tightly coupled. When taking the solution from the glass bottle, a part of GLM with a plastic tube with glucose needs to be very carefully carried in a vertical position, and solutions in the glass bottle and in the plastic tube must be maintained at a constant level throughout the entire study; however, it is only necessary to make up the fluid to a predetermined amount/volume at each measurement. Finally, performing the test requires cooperation with a chemical laboratory which uses specialized apparatus (e.g., a spectrophotometer), and all samples must be stored under appropriate temperature and humidity conditions [22, 23]. The Shemesh modification was used in this study to avoid rapid evaporation of fluid/glucose [23].

Despite its limitations and its labor- and time-consuming character, the glucose penetration test has been used by many researchers [22, 23, 26, 27]. Its great advantage is also that leakage measurements can be made at any time intervals determined by the investigator. Measurements of the glucose filtrate can be performed several times at selected intervals during the study period, or at only one point: for example, several weeks or several months from the start of the experiment. However, in the opinion of the authors of the present study, a few measurements should be taken to allow a better evaluation of filling quality. The use of frequent measurements gives a better picture of the quality of the root canal fillings, allows the investigator to accurately track the glucose leak, and best realizes the potential of the method.

In this study, a slow, gradual increase in glucose leakage was observed over the 13 measurements performed during the experiment. No statistically significant differences were found in the degree of sealing ability of root canal fillings (*p* > 0.05). Similar results were achieved by Kececi et al. [27], who report that lateral condensation and continuous wave compaction (System B + Obtura II®) are comparable sealing methods. The first and the last measurement of glucose concentration was performed three months after the commencement of the experiment. In another study, slightly higher glucose concentration was observed in canals filled using the lateral condensation technique than those obturated with the thermal method (System B + Obtura II) during the first eight days of the experiment; however, no significant difference in the seal of canal fillings was noted after the second week of observations [28]. Xu et al. [29] found worse long-term seals when using the lateral condensation technique than thermal methods (continuous wave compaction and the technique with thermoplasticized Thermafil® obturator). No statistically significant difference was found between the lateral condensation thermal methods with regard to sealing ability, but only for the first two weeks of the study. From the second to the twelfth week of the experiment, a lower glucose concentration was recorded in the group of teeth filled with thermal methods. At the same time, no significant difference was found between the two methods regarding the number of teeth without tracer leakage [29].

Long-term evaluation of the seal of root canal fillings was also carried out using fluid filtration and bacterial tests [16–18, 20, 30–33]. After a 16-month specimen incubation, no significant differences were observed between the lateral condensation technique of cold gutta-percha and the vertical compaction of warm gutta-percha (System B + Obtura II) regarding their ability to seal fillings [16]. Higher values of fluid leakage were observed after using the Epiphany® and Resilon root canal filling materials compared to gutta-percha and AH Plus sealer [16]. Similar seals were found in canals filled with the cold gutta-percha lateral condensation technique, the thermal technique with plasticized obturator (Thermafil) and warm vertical compaction (System B) during a two-month study of bacterial penetration [30]. Similar results were obtained after one and four months of the bacterial test. A comparable passage of bacteria was reported in the canals filled with the cold gutta-percha lateral condensation, vertical compaction of warm gutta-percha, continuous wave compaction, and injection methods [19–21]. Gencoglu et al. (2007) [18] report lower fluid penetration in canals filled with thermal methods (Thermafil, Soft-Core®, or System B) as compared to the canals filled with the cold gutta-percha lateral condensation technique after two-year incubation of specimens using a zinc oxide-based sealer and eugenol (Kerr Pulp Canal Sealer®). Because the teeth were stored for two years in a moist environment, that is, during the incubation period, the plastic material might have partially dissolved in the group of canals filled by lateral condensation, in which a greater amount of sealer is usually applied than in thermal techniques [18]. On the other hand, some studies indicate that a worse seal was achieved after using the thermal method (Touch'n Heat + Obtura II) than when cold gutta-percha was used [20]. This difference may be due to the type of root canal sealer used: polydimethylsiloxane based root canal sealer, which undergoes faster polymerization under the influence of high temperature. The impairment of silicone bonds and shortened binding time hinder the penetration of the material into irregularities of the pulp cavity and prevent a tight connection being made with the gutta-percha [20].

## 5. Conclusion

The lateral condensation of cold gutta-percha can guarantee a similar seal of canal fillings as can be achieved by using thermal methods, in the round canals. The glucose leakage test is a suitable long-term method to evaluate the sealing ability of root canal fillings.
